# An Innovative Biofunctional Composite Hydrogel with Enhanced Printability, Rheological Properties, and Structural Integrity for Cell Scaffold Applications

**DOI:** 10.3390/polym15153223

**Published:** 2023-07-28

**Authors:** Taufik Abdullah Mappa, Chung-Ming Liu, Chung-Chih Tseng, Muhammad Ruslin, Jui-Hung Cheng, Wen-Chien Lan, Bai-Hung Huang, Yung-Chieh Cho, Chia-Chien Hsieh, Hsin-Hui Kuo, Chen-Han Tsou, Yung-Kang Shen

**Affiliations:** 1 School of Dentistry, College of Oral Medicine, Taipei Medical University, Taipei 110, Taiwan; d204111004@tmu.edu.tw (T.A.M.);; 2 School of Dental Technology, College of Oral Medicine, Taipei Medical University, Taipei 110, Taiwan; m225098012@tmu.edu.tw; 3 Department of Oral and Maxillofacial Surgery, Faculty of Dentistry, Hasanuddin University, Makassar 90245, Indonesia; mruslin@unhas.ac.id; 4 Department of Biomedical Engineering, College of Biomedical Engineering, China Medical University, Taichung 404, Taiwan; liuc@mail.cmu.edu.tw; 5 Institute of Medical Science and Technology, National Sun Yat-sen University, Kaohsiung 804, Taiwan; caviton@gmail.com; 6 Department of Mold and Die Engineering, National Kaohsiung University of Science and Technology, Kaohsiung 807, Taiwan; rick.cheng@nkust.edu.tw; 7 Department of Oral Hygiene Care, Ching Kuo Institute of Management and Health, Keelung 203, Taiwan; jameslan@ems.cku.edu.tw; 8 Graduate Institute of Dental Science, College of Dentistry, China Medical University, Taichung 404, Taiwan; u109312001@cmu.edu.tw; 9 Research Center for Biomedical Devices and Prototyping Production, Taipei Medical University, Taipei 110, Taiwan; d225103001@tmu.edu.tw; 10 Department of Dentistry, Zuoying Branch of Kaohsiung Armed Forces General Hospital, Kaohsiung 813, Taiwan

**Keywords:** biopolymer, hydrogels, printability, cell scaffold, tissue regeneration

## Abstract

The present study was conducted to manipulate various biomaterials to find potential hydrogel formulations through three-dimensional (3D) bioprinting fabrication for tissue repair, reconstruction, or regeneration. The hydrogels were prepared using sodium alginate and gelatin combined with different concentrations of Pluronic F127 (6% (3 g), 8% (4 g), and 10% (5 g)) and were marked as AGF-6%, AGF-8%, and AGF-10%, respectively. The properties of the hydrogels were investigated using a contact angle goniometer, rheometer, and 3D bioprinter. In addition, the osteoblast-like cell line (MG-63) was used to evaluate the cell viability including hydrogels before and after 3D bioprinting. It was found that the ratio of contact angle was lowest at AGF-6%, and the rheological results were higher for all samples of AGF-6%, AGF-8%, and AGF-10% compared with the control sample. The printability indicated that the AGF-6% hydrogel possessed great potential in creating a cell scaffold with shape integrity. Moreover, the live/dead assay also presented the highest numbers of live cells before printing compared with after printing. However, the number of live cells on day 7 was higher than on day 1 before and after printing (** *p* < 0.01). Therefore, the combination of AGF-6% could be developed as a biofunctional hydrogel formulation for potential tissue regeneration applications.

## 1. Introduction

The demand for bone graft substitutes is increasing worldwide due to the increasing success of post-replacement grafts [1]. The occurrence of bone defects due to injury, diseases, or surgery causes loss of function and requires treatment in the form of tissue or organ transplantation. In the last decades, autografting and allografting have functioned as the gold standard for bone regeneration; however, there are several limits, including the existing suitable bone tissue that is obtained from an organ donor, rejection by the immune system, and eternal pain at the donor site after surgery procedures [2,3]. Because of the limitations of organ donors, three-dimensional (3D) bioprinting is a concrete solution to the reconstruction of organs for tissue engineering, and they can be quickly printed without organ donation [3]. The combination strategies of tissue engineering between biomaterials and the progenitor of cells are an alternative to developing bone grafting methods for bone tissue regeneration [2]. Despite remarkable research efforts, 3D bioprinting is a rapid prototyping technology that creates 3D scaffolds layer-by-layer to repair bone defects, and the technique is also precise enough for the multi-layer construction of different complex cell types and tissue models; the primary approach is to seed and grow cells in 3D scaffolds that promote print precision, cell differentiation, and bone formation in vitro for transplantation [4,5].

Hydrogels composed of multiple biomaterials have also been used in filament construction, and this is one of the greatest challenges for hydrogels that needs to be resolved [6,7,8]. The viable hydrogels should possess certain properties, such as excellent structural stability, biocompatibility, and support of mechanical and rheological characteristics [7,9,10]. The combination of hydrogel materials should be considered as an important factor in the design quality of 3D bioprinting in order for the printability and cell viability function to have excellent results [11,12,13]. Generally, the printed filament has been designed depending on the critical size of bone tissue defects, and the probability of the size is different. Additionally, filament collapse affects the stability of each layer. The filament structure should be precisely designed with the biological architecture and organ shape, and the printability design needs to have high resolution and shape fidelity. The printability concept depends on the ability of 3D structures to be formed with integrity and a compatible fidelity. This represents the critical property where the organ and bone defects are required. In addition, the function and geometry of the filament are crucial, and several demonstrations of the filament structure are based on printing techniques. The 3D bioprinting-based micro-extrusion approach has the capability of creating 3D filaments with controllable parameters and morphology design in the structure formation. However, it is mostly important to focus on the formulation of bioinks to assess the printed scaffold, rheological behavior, and cell compatibility [1].

The 3D bioprinting of hydrogels can be printed immediately using cell suspension or cell-free hydrogels [13]. By incorporating developmental cell biology, regenerative medicine, and bio-tissue engineering, 3D bioprinting provides a platform to artificially repair accurate tissue mimics [4]. Some varieties of natural polymers are applicable for three-dimensional bioprinting, including gelatin, alginate, collagen, HA, fibrin, and chitosan [13]. The materials of hydrogels that are obtained from natural polymers are the type of bio-macromolecule aqueous solutions whose source is from plants or animals; for example, gelatin and alginate composites are often used to improve mechanical properties and biocompatibility [7]. Alginate and gelatin composite hydrogels are the most widely used composites for fabricating tissues and organs for bone engineering; they have cell-binding motifs to facilitate cell adhesion, excellent structural integrity, and extrusion printability [14,15]. Due to the latter, exploring the printability of irregularly shaped hydrogels is necessary to improve the rheological properties of alginate–gelatin hydrogels and ensure good printability, shape fidelity, and cell viability [16,17]. When designing biopolymer inks, one of the most important considerations is the rheology characterization. Rheological tests of biopolymer inks are widely used as a standard measurement for the optimization of processing; they also provide information about the parameters of the polymer structure in heterogeneous systems and are necessarily focused on hydrogel viscosity to supply the rheological properties [18]. The rheological behavior of natural polymers is quite tricky due to the viscoelasticity and shear properties having their own role. The strong characteristics of viscoelasticity are influenced by temperature and air pressure. Meanwhile, other properties cannot be guaranteed to entirely derive from the shear behavior. Moreover, the chemical composition of macromolecular biopolymers is almost variable, which is mainly related to the viscosity and shear properties due to their suitability for analytical studies [18].

In this study, Pluronic F127 was selected as the synthetic polymer for combining the alginate–gelatin composite, which has the capability of acting as a cell carrier, has a low modulus and toxicity, and has thermoregulation reversibility that displays the rheological behavior as an innovation to improve the printability of 3D scaffolds [7,17,19,20,21,22]. However, Pluronic F127 lacks cell binding when not combined with other biomaterials and rapidly dissolves under high temperatures, which impedes its use as a bioink [20]. Meanwhile, alginate hydrogels are created as scaffolds due to the degradation kinetics being tailorable, the ease of gelation, and their biofunctionalization being cell-laden; sodium alginate was also selected due to it being mostly applicable for 3D scaffold cell culture [2]. In general, it does not work for 3D free structures that form without any active cure during the printability of objects or that modify the rheological properties as additional methods [20]. While gelatin from natural polymers is often used for 3D bioprinting due to its easy formation, biodegradability, and excellent biocompatibility, it has poor mechanical strength and fast degradation in the human body [10,15,23]. However, developing the mechanical properties of hydrogels that are required for a high printing resolution with respect to shape fidelity while maintaining the viability of the cell representation of Pluronic F127 can accelerate the conformational change due to the interaction between alginate–gelatin under a temperature of 40 °C (following the concentration), which affects the performance of various rates of viscosity and the ability to simply print without excessive air pressure for the cell encapsulate [22,23]. In addition, the reconstruction of the multi-layer filament structure demands high-resolution printability and the prevention of collapse after printing [15,20].

Through the incorporation of other materials, it is possible to modify the alginate–gelatin composite with the addition of Pluronic F127 to make it more suitable and enhance the mechanical strength and stability of the hydrogel properties [23,24,25]. Therefore, the present study aimed to investigate the combination parameters of sodium alginate, gelatin, and Pluronic F127 as a potential hydrogel by assessing their rheological properties, printability, and cell viability, which will allow for the future development of customized 3D bioprinting cell scaffolds for the applications of bone and tissue regeneration.

## 2. Materials and Methods

### 2.1. Hydrogels Preparation

Sodium alginate (187 kDa, W201502), gelatin from porcine skin (50–100 kDa, gel strength 300 type A, G2500), and Pluronic F127 (P2443) were purchased from Sigma Aldrich (Taipei, Taiwan). Hydrogel formulations were prepared using a 6% concentration of alginate (3 g) and 1% concentration of gelatin (0.5 g), which were dissolved into 50 mL of phosphate buffered saline solution (PBS, Sigma Aldrich, Taipei, Taiwan) and then stirred using a mechanical stirrer (Chemist, MS-1400D, New Taipei City, Taiwan) at a temperature of 65 °C for 1 h. Then, Pluronic F127 was added with different concentrations (6% (3 g), 8% (4 g), 10% (5 g)) into the alginate and gelatin solution in an ice bath under a temperature of 4 °C for 1 h. We centrifuged the hydrogel hydrogels for 5 min at 21 °C with 2500 rpm and kept the mixture at a temperature of 4 °C. We prepared 100 mM of crosslinking agent by incorporating dissolved calcium chloride dihydrate (CaCl_2_) (Sigma Aldrich, C7902, Taipei, Taiwan) into the phosphate buffered saline solution. The hydrogels were set at room temperature for 4 h. The resulting hydrogel is denoted according to the concentration of Pluronic F127 added, namely, AGF-6%, AGF-8%, and AGF-10%. The combined alginate and gelatin hydrogel without a Pluronic F127 addition was used as a control group, which is the AG sample.

### 2.2. Contact Angle Testing

The hydrophilicity behavior of hydrogels was measured using contact angle testing and was evaluated using a pure substrate of bioink. We dropped 0.05 mL of hydrogel onto a glass surface with 10 mm of absolute distance under a control time at 0, 2, 4, 6, 8, and 10 min; then, the contact angle was measured using the GBX Digidrop goniometer (GBX Scientific LTD., Romans-sur-Isère, France). We adjusted the temperature to 25 °C and maintained the humidity assessment at 60%, respectively.

### 2.3. Rheological Measurement

The analysis of the rheological properties for each hydrogel was carried out using an HR 20 Discovery hybrid rheometer (TA Instruments, Taipei, Taiwan) that was equipped with a 25 mm to 40 mm diameter plate with ceramic insulation and a DIN concentric cylindrical rotor with a diameter of 28 mm. Using a gap-measuring position resolution of 0.02 µm, we carried out the flow rate sweep under a shear rate ranging from 0.01 to 500 s^−1^ at 25 °C.

### 2.4. Filament Fusion Analysis

A self-assembled micro-extrusion-based 3D bioprinter with a 22-gauge needle (0.4 mm diameter nozzle) was applied to investigate the printability of the hydrogels. The hydrogels were extruded at different air pressures (2.0, 2.2, 2.4, 2.6, and 2.8 bar) to print a pattern at a size of 10 mm^2^ (at a vertical distance of 4, 3, 2, and 1 mm (1st layer) and horizontal gap of 1, 2, 3, and 4 mm (2nd layer), respectively). In addition, the hydrogel was extruded using a print speed of 5 mm s^−1^ and under a temperature of 25 °C. To prevent dimensional changes in the scaffold, the filament was imaged directly after it was printed using a phone camera with a high resolution, and it was compared with each sample.

### 2.5. Filament Collapse Analysis

The platform of filament testing has five pillars in the middle with the dimensions 2 × 10 × 6 mm^3^; the right and left side ends of the pillar are 5 × 10 × 6 mm^3^, and we controlled the space to be 1, 2, 3, 4, 5, and 6 mm between each pillar. Formlabs Inc. (Somerville, MA, USA) was used as a platform tester. The 3D printing print speed was 5 mm s^−1^, and it used a 0.4 mm diameter gauge nozzle. The constant air pressures of AGF-6%, AGF-8%, and AGF-10% were extruded at 2.0, 2.2, 2.4, 2.6, and 2.8 bar, respectively, under a temperature of 25 °C, where we then immediately photographed the filament after extrusion using a camera with a high resolution.

### 2.6. Multilayer Stability Assessment

The multilayer stability assessment of the investigated hydrogels was printed in 10 layers (10 × 10 × 3.5 mm^3^) using a self-assembled 3D bioprinter with an extruded syringe dispenser system under an air pressure of 2.6 bar, printing speed of 5 mm s^−1^, and control time of 0, 4, 8, and 12 min. The images of the multilayer stability test were acquired using the GBX Digidrop goniometer connected to Visidrop^®^ software. The dimensional change was measured by drawing a line from the lowest left corner to the mid-highest point of the 10th layer filament and then to the lowest right corner of the filament, thus forming a triangular line.

### 2.7. Evaluation of Cell Viability

**Figure 1:**
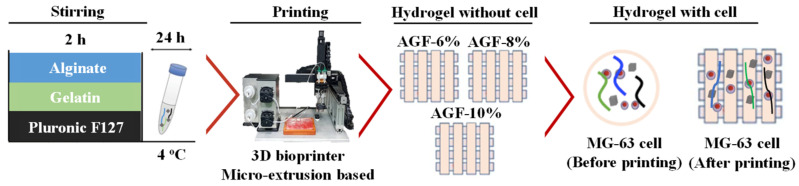
The schematic of the 3D bioprinting process for evaluating the properties of the investigated hydrogels.

The MG-63 cell suspension (4 × 10^5^ live cells/mL) with 1 mL of hydrogel was extruded (used 5 mL syringe) into a dish (35 mm); we added 3 mL of CaCl_2_ solution at a concentration of 100 mM from the PBS solution as a crosslinking agent and then soaked the suspension for 30 min to evaluate the MG-63 cell viability within the hydrogel before printing treatment. After that process, the crosslinking agent solution was released and washed using PBS solution three times. We added approximately 2 mL of culture medium (DMEM/F12) and then incubated the hydrogels at 37 °C with 5% CO_2_. Meanwhile, the post-printing cell viability was conducted using 1 mL of the hydrogel and was printed into a dish with 4 × 10^5^ live cells/mL of MG-63 cell. The printer temperature and air pressure were 25 °C and 2.6 bar, respectively. The printed hydrogel with MG-63 cell was soaked in the same crosslinking agent solution for 30 min. After that, the crosslinking agent solution was released and washed using PBS solution three times, and 2 mL of DMEM/F12 was then incubated at 37 °C with 5% CO_2_. The cell viability analysis was performed using LIVE/DEAD^®^ cytotoxicity kit fluorescent dyes (488/570; Thermo Fisher Scientific, Paisley, UK), and the cells were incubated at 37 °C and 5% CO_2_ for 1 h. After 1 h of live/dead staining procedures, the cells were investigated using an inverted microscope. For each experiment, we examined a nuclear field from the hole at different magnifications. Cell viability was observed before printing on days 1 and 7 and after printing on days 1 and 7. By using the VisiView software (Visitron Systems, Puchheim, Germany) to measure the cells and by displaying dead cells in red color and live cells in green color, we then counted 3 images, which have been randomized to calculate the live and dead cells using Image J image processing software (version 1.53t). A schematic diagram in Figure 1 displays the performance of the experimental procedures applied to evaluate the cell viability pre-printing and post-printing along with the hydrogels.

### 2.8. Statistical Analysis

The data analysis was presented as mean ± SD using the SPSS statistic package (19.0 Version., SPSS Inc., Chicago, IL, USA). The differences between multiple groups were analyzed using a one-way test of variance followed by an analysis using Tukey’s HSD post hoc method. The data significance was defined as * *p* < 0.05 and ** *p* < 0.01.

## 3. Results

### 3.1. Wettability Variation

**Figure 2:**
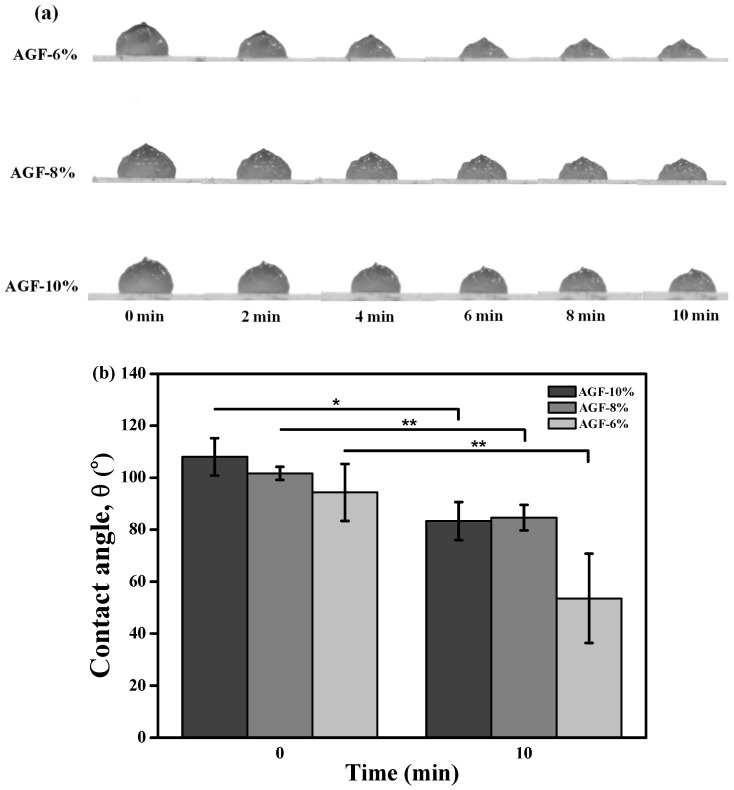
(**a**) wettability results of AGF-6%, AGF-8%, and AGF-10% and (**b**) the quantitative measurement analysis (* *p* < 0.05 and ** *p* < 0.01).

The wettability demonstrated in the concentration for each group of AGF is presented in the hydrophilic characteristics after dropping the 10th min of hydrogel, as shown in Figure 2. As mentioned in Figure 2a, the hydrogel droplets for each sample performed the collapse, which resulted during the time of observation on the glass plate. The droplet inks changed the dimensionality and shape of the hydrogel. When a hydrogel that is extruded through the contact angle rapidly transforms the dimensionality, this means that the inks have solid or liquid characteristics. As found in our result, the greatest dimensional change occurred in the AGF-6% group, which was about 38% collapsed after the 10th min was observed (Figure 2b). However, the loss rate can be different between the contact angle and 3D bioprinting instruments due to the air pressure and print speed not being applied when the hydrogel is pumped through the contact angle analysis.

### 3.2. Rheological Property

**Figure 3:**
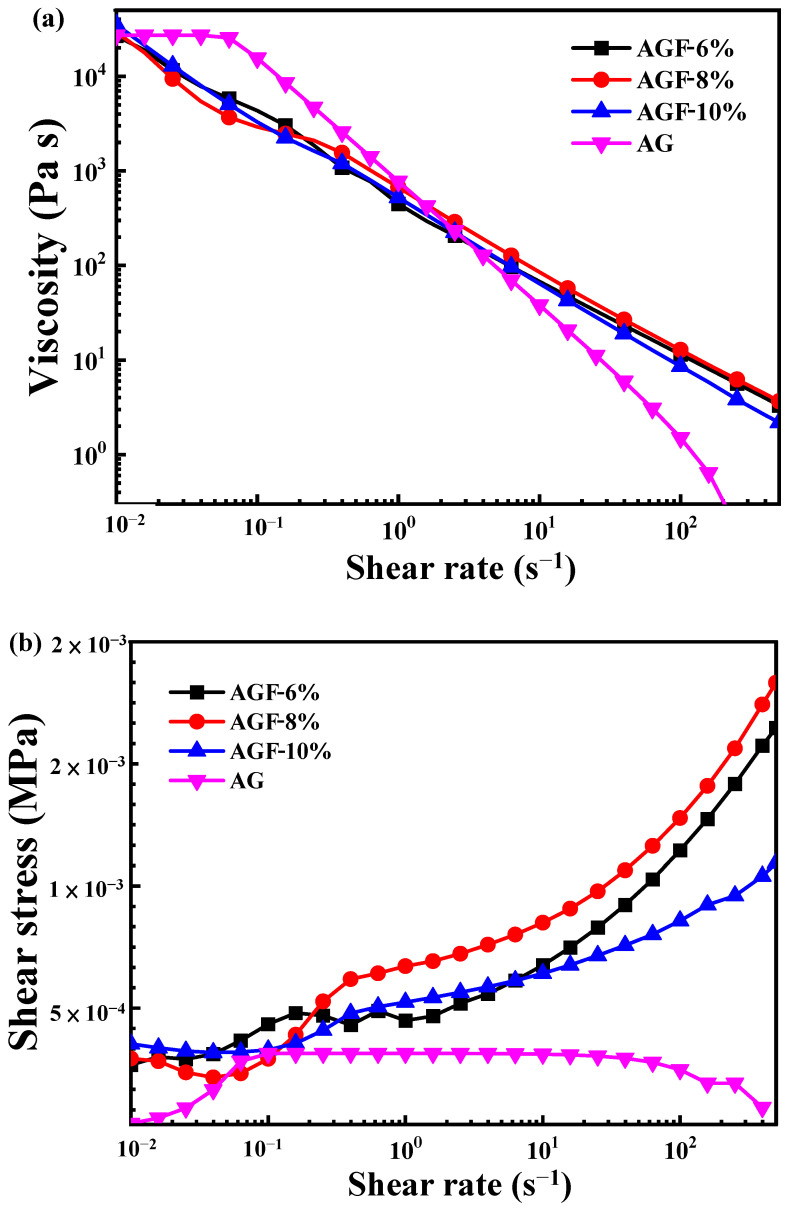
The (**a**) viscosity and (**b**) shear stress of the investigated hydrogels.

The rheological behavior of the investigated materials used in this study, namely sodium alginate, gelatin, and Pluronic F127, was measured using a standard protocol. Each material was tested with different concentrations to observe the concentration of hydrogel effects in terms of rheological variations. Briefly, natural and synthetic biomaterials that are based on their viscoelasticity characteristics for use as hydrogels should be biocompatible and easy to extrude through a thin nozzle. The comparison between the rheological properties of alginate, gelatin, and Pluronic F127 under different concentrations was performed. Improving the concentration of Pluronic F127 influenced the viscosity properties and shear stress value (Figure 3). When compared with the hydrogel without Pluronic F127 as a control, the viscosity property showed little resemblance with AGF-6%, AGF-8%, and AGF-10% having more than 10^4^ Pa s, and they had a low shear stress rate of under 2 × 10^−4^ MPa.

### 3.3. Printability Characterization

**Figure 4:**
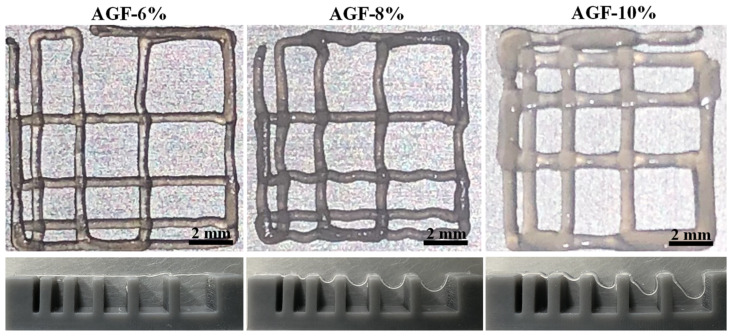
Filament fusion (upper line) and filament collapse (bottom line) results of the investigated hydrogels under an air pressure of 2.6 bar.

**Figure 5:**
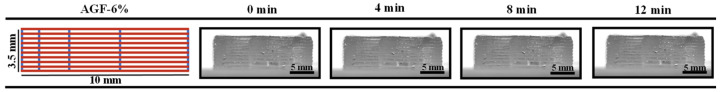
The 10th layer printability of the optimal hydrogel AGF-6% for multilayer stability testing.

After the filament fusion and collapse testing (Figure 4), the results displayed the filament fusion of AGF-6%, AGF-8%, and AGF-10% as having printed the completed wire pattern at an air pressure of 2.6 bar. The collapse testing of AGF-6% showed that the filament extruded the hydrogel well into a line of the platform. A similar result for AGF-6% can also be found in the control group for the filament testing [15]. The extrudability of uniforms also printed the 10th layer of the filaments (Figure 5). From zero to twelve minutes, the loss thickness of the AGF-6% hydrogel was found to be a negative result. This phenomenon is related to the integrity of the structure. As the viscosity increases, the printed structure negatively affects the loss of shape. This then confirms the hypothesis that a high-viscosity hydrogel will negatively impact the fidelity of filament shape in printability. The smooth surface of the printed structure has no effect on the viscosity values.

### 3.4. Cell Viability and Response

**Figure 6:**
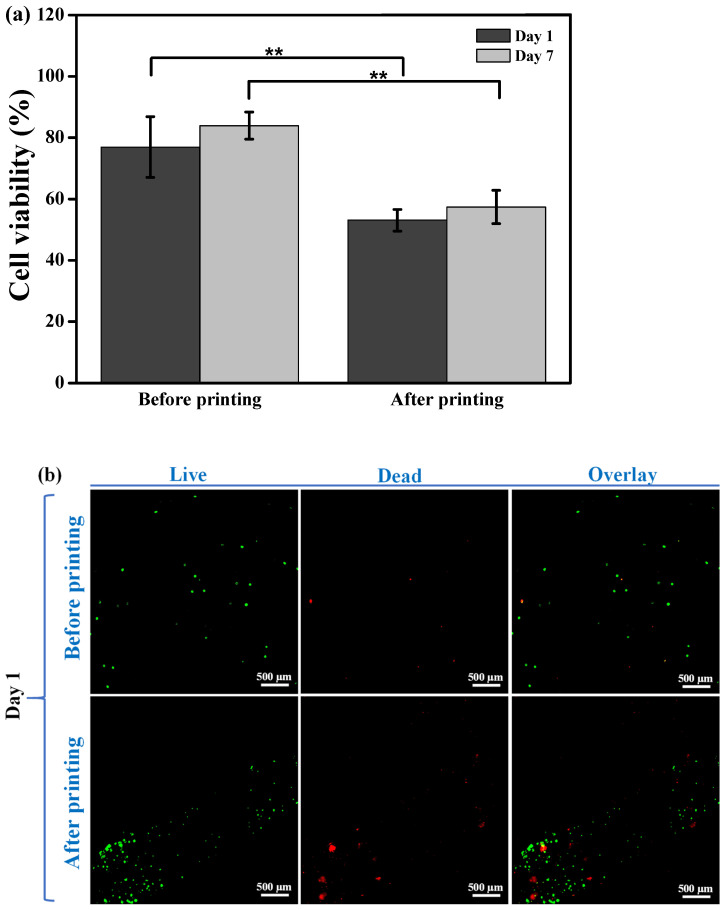

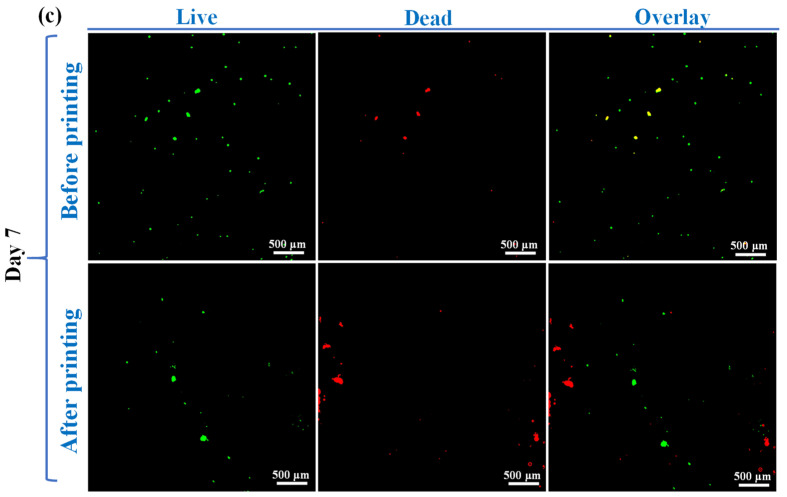
Cell viability results of the optimal hydrogel AGF-6% through a live/dead analysis: (**a**) quantitative measurement analysis (** *p* < 0.01), (**b**) day 1, and (**c**) day 7.

Figure 6 displays the cell viability staining analysis of the AGF-6% hydrogel. The cell viability of the AGF-6% demonstrated the highest numbers of cell viability before printing was performed compared with after printing (Figure 6a). The statistics showed different amounts for before and after printing on day 1 and day 7 with ** *p* < 0.01. The beginning section has explained the decrease in the number of cell viability after printing (Figure 6b) compared with before printing (Figure 6c). However, the number of live cells on day 7 was higher than on day 1 both before and after the printing treatment. This result showed that the cell viability increased in the cells that were extruded after day 7 using the MG-63 cell line. Moreover, this supports the hypothesis that the cell viability for AGF-6% with MG-63 cell will advance from day to day with respect to the number of cells that live.

## 4. Discussion

The ability of printability hydrogels can be defined by several parameters, but this assessment lacks standardized methods as to which are the best choices in an analysis [2,15,26]. The parameters of printability are often evaluated using rheological properties tests such as viscosity, shear stress, and stability assessment to identify the optimal printability parameters for the application [27]. The characterization of hydrogel shape fidelity can also be analyzed by the minimum hydrogel extrusion that is used in the contact angle measurement but cannot be a standard printability method for the dimensional stability of hydrogels. The functionalization of hydrogels after extrusion is required to ensure the deformation of the physical filament and the degree of similarity to the initial design [2,26]. In this experiment, Pluronic F127 was chosen as a synthetic biomaterial for the hydrogels due to the Herschel–Bulkley fluid principles, and it is great in 3D bioprinting applications for establishing low viscosity and shear rate properties during printing [20]. Alginate–Pluronic F127 or gelatin–Pluronic F127 hydrogels are often used for bioprinting to create 3D structures due to their thermo-gelation reversibility and good printability [28]. However, the mixture of three biomaterial polymers such as sodium alginate, gelatin, and Pluronic F127 as AGF groups for cell binding and solidity characteristics, due to the thermo-reversibility characteristic of Pluronic F127, is difficult to combine together during preparation as a hydrogel. In this study, the viscosity of the AGF-6% sample was similar to the results of the previous study as a control group, which means it is good for printability [2,22]. The viscosity value, which was almost the same as the AG group, was influenced due to the low concentration of Pluronic F127 in the AGF-6% sample. In contrast, the shear stress of the AGH-6% sample was lower than the AGF-8% and AGF-10% samples. Meanwhile, the control group had mostly lower values than other samples. In summary, the concentration of Pluronic F127 6% had viscosity stability even though it was combined with sodium alginate and gelatin. However, the strategies to reduce the shear stress could be adjusted for in the composition of each material (gelatin and sodium alginate) in further studies. Hydrogels with high shear stress impact the death of cells in the tissue printing process [20]. On the other hand, contact angle measurement as a printability test cannot be a standard for the dimensional stability of hydrogel without accounting for the print speed and air pressure parameters [29].

Our study conducted filament fusion and collapse testing using an air pressure of 2.0, 2.2, 2.4, 2.6, and 2.8 bar to measure the hydrogel shape fidelity after printing and investigate the optimal air pressure for each concentration [26,29]; we obtained the optimal printability for each hydrogel to be at 2.6 bar of air pressure. The increase in air pressure was caused by the addition of Pluronic F127 into the alginate and gelatin solutions, resulting in a high shear stress rate, which required greater air pressure to extrude the hydrogels. The latest study from Peng et al. [15] explained the printability of the control group hydrogel, which printed the filament at lower air pressures than the AGF-6% sample; this was possibly due to ease of gelation kinetics that were caused without the additional inclusion of Pluronic F127 [2,12]. Furthermore, the viscoelasticity rate that is related to the extrusion of hydrogel affects the stability shape of the filament [17,30,31]. Hydrogels with low viscosity can maintain the spread of hydrogels, but when the rate of viscosity is too high, then high air pressure is required to encourage the hydrogels that disrupted the cell viability [17,32,33,34].

Understanding the mechanisms of the rheological properties that changed the cell environment of hydrogels for 3D bioprinting tissues remains the focus of research [20]. The evaluation of in vitro studies during the printing of hydrogels requires the maintenance of cell viability before and after the printing treatment. However, the post-printing cell viability depends on the hydrogel composite and the shear stresses that are produced for the printing process [2]. On day 1 and 7 before printing, the cell viability was more than 70%. Meanwhile, on day 1 and 7 after the printing treatment (10 mm diameter by 0.35 mm height), the cell viability decreased. This difference can be caused by the use of a large air pressure (2.6 bar) to extrude the AGF-6% hydrogel. Compared with the report by Gao et al. [15], it was found that the post-printing cell viability was higher in the control hydrogel than in the AGF-6% hydrogel. This difference is in line with the principle of viscoelasticity. Hydrogels produced with a higher viscosity and extruded air pressure will affect the number of cells extruded. The utilization of air pressure through 3D bioprinting can predict the cell viability numbers. One research report from Reina-Romo et al. [35] explained that air pressure and the high rate of shear stress were primary causes of mortality in hydrogel composite cells. In the present study, the solid characteristics of the AGF-6% hydrogel impacted the rheological properties as well as the cytocompatibility. The cell viability results of the AGF-6% hydrogel exhibited a decrease in the number of cells at less than 70% on day 7 after bioprinting. On the other hand, the cell viability increased compared with day 1 after bioprinting. This feature means that the post-printing cell viability of MG-63 cells in the potential AGF-6% hydrogel could be increased with an incubation period of cells over 7 days. Nevertheless, further experiments must be conducted to evaluate the long-term period of post-printing cell viability in the potential AGF-6% hydrogel and to determine an optimal concentration of Pluronic F127 for cell scaffold applications.

## 5. Conclusions

The combination of sodium alginate, gelatin, and Pluronic F127 as hydrogels showed the high rheological properties of all samples compared with the control sample. The filament collapse and fusion testing results found an optimal air pressure of 2.6 bar for the AGF-6% hydrogel printing. After the 10th layer of printing, the AGF-6% hydrogel possessed a filament with good stability and shape integrity. The cell viability assay also exhibited an increased cell number in the AGF-6% hydrogel 7 days post-printing. Accordingly, these findings demonstrate that the AGF-6% hydrogel could be developed as a potential cell-laden hydrogel to print cell scaffolds with cell viability and structural integrity for tissue engineering applications.

## Data Availability

The data are contained within the article.
